# A novel protocol for the characterization of ureteral stent echogenicity

**DOI:** 10.1186/s12894-025-01931-3

**Published:** 2025-09-02

**Authors:** Clifford Jiajun He, Tyler Sheetz, Jonathan Katz, Vivek Pendem, Kevin Ho, Nicholas Trujillo, Sujit Tunuguntla, Robert Sah, Seth K. Bechis, Roger L. Sur, Manoj Monga

**Affiliations:** 1 Department of Urology, School of Medicine, University of California, 9300 Campus Point Drive, La Jolla, San Diego, CA 92037 USA; 2 Jacobs School of Engineering, University of California, San Diego, CA USA

**Keywords:** ureter, stent, biomaterial, ultrasound, echogenicity, silicone

## Abstract

**Background:**

Decreasing radiation exposure in renal colic management involves revisiting diagnostic, intraoperative, and postoperative practices. One approach is a shift in procedural standards from intraoperative fluoroscopy to ultrasonography. Despite the increased implementation of ultrasound in urology, the ultrasound visibility or echogenicity of various stents is not well-studied. Silicone stents, traditionally considered less radiopaque, pose challenges during placement under fluoroscopy due to their reduced visibility. However, a shift to ultrasound-based guidance can levels the playing field, as silicone stents demonstrate comparable echogenicity to stents made from other materials. This study aims to evaluate and quantify the echogenicity of different ureteral stents on the market, with a focus on silicone stents, which traditionally have been considered less radiopaque yet potentially advantageous for less patient discomfort.

**Methods:**

We conducted a simulation study using five different brands of ureteral stents in an ex-vivo porcine bladder model. We measured the mean grey intensity to quantify echogenicity of each stent. After comparing the echogenicity of individual stents, we grouped stents based on materials and diameters and performed T-tests.

**Results:**

We established that ureteral stents can demonstrate good visibility inside porcine bladder under ultrasound. Upon further investigation, we found that diameter is the most important variable in determining the measured echogenicity of ureteral stents. 8 Fr ureteral stents have higher measured echogenicity than the Sensor wire and 6 Fr stents. Stent materials also play a role in the measured echogenicity of ureteral stents. With the same diameter, silicone is measurably more echogenic than polyurethane.

**Conclusion:**

This study describes a novel quantitative assessment of ureteral stent echogenicity, which can guide the development of novel echogenic stents and provide a benchmark for existing stents. In addition, it suggests that ultrasonography may facilitate the placement of silicone stents, which historically have been more difficult to visualize with fluoroscopy.

## Introduction

The modern-day double-J ureteral stent was introduced in 1978 by Finney et al. [1], and it has since become one of the most common and essential tools in urology. Despite their widespread use, ureteral stents have been associated with an array of complications, including infections, encrustations, and patient discomfort [2]. Stent colic, or renal colic attributable to an indwelling ureteral stent, is the most common reason for emergency room visits after kidney stone procedures [3]. Concurrently, ureteral stents have undergone significant innovations aimed at enhancing patient outcomes by minimizing discomfort and reducing encrustation [4]. 

Currently, the most common ureteral stents are made of polymeric materials, mainly polyurethane or silicone [4, 5]. Stent diameters range from 6 to 8.5 Fr, with some studies suggesting smaller diameters cause less encrustation [6, 7]. To minimize patient pain and discomfort, polymeric materials with higher flexibility are in development to improve patient quality of life [8]. Metal is another material used to construct ureteral stents, and their durability enables them to stay functional longer in patients [9]. Biodegradable ureteral stents are another promising development that could eliminate the need to remove them from patients post-treatment and reduce the frequency of surgery. Besides material, another future direction is the modification of stent coating. Hydrogel is a material that becomes more slippery when in contact with water, reducing the friction of stent insertion. Coating with ketorolac, a nonsteroidal anti-inflammatory drug, reduces the pain of insertion. Antibiotic or metal compound coating reduces the formation of biofilms and encrustations [8]. 

In recent years, reducing radiation exposure has become a critical consideration in the management of patients with renal colic. Traditional intraoperative imaging often relies on fluoroscopy, which, while effective, exposes patients to ionizing radiation and fails to image soft tissue structures, overlooking an important safety mechanism. In response, ultrasound has played an increasingly integral role in diagnosing and treating urological symptoms due to its ease of use, reduction of complications, and minimization of radiation [10]. However, the echogenicity, or ultrasound visibility, of different stents remains underexplored. This study introduces a novel quantitative methodology to assess echogenicity by measuring mean grey-scale intensity with ImageJ software. This provides a standardized, replicable, and free-of-charge protocol to evaluate stent visibility under ultrasound, addressing a current gap in the literature.

The objective of this study was to fill this knowledge gap by evaluating the echogenicity of different ureteral stents available in the market and assessing the possibility of quantifying their properties with measured echogenicity. In particular, silicone stents may afford advantages with regard to stent comfort and encrustation, yet they have traditionally been less radiopaque, which can make fluoroscopic placement more challenging [11–13]. However, a shift to ultrasound-based guidance may level the playing field by providing comparable visibility for silicone stents relative to other stent materials. By leveraging ultrasound’s ability to enhance the visibility of stents irrespective of their radiopacity, we hypothesize that ultrasonography can not only reduce radiation exposure but also facilitate the placement of stents. This study aims to evaluate the utility of ultrasonography to overcome this limitation in utility.

## Methods

### Clinical trial registration

This study is not a clinical trial and does not require registration.

### Study design

We conducted a simulation study using five different brands of ureteral stents in an ex-vivo porcine bladder model. The study employed ultrasound recordings with a Butterfly iQ + probe, using a standard Sensor wire as the baseline reference. The Sensor wire was chosen because it is a standard and consistently visible instrument in endourologic procedures, which is a stable baseline for echogenicity comparison for this study.

### Materials

The ex-vivo porcine bladder used in this study was obtained from the laboratory of Dr. Robert Sah at the University of California, San Diego. No live animals were used in this study. As shown in Table 1, the five ureteral stents with their diameters and materials were Cook Medical Black (6 Fr silicone), Bard Fluoro (6 Fr silicone), Coloplast Imajin Hydro (6 Fr silicone), Coloplast Stenostent (8 Fr silicone), and Boston Scientific Ascerta (6 Fr polyurethane). The Coloplast Stenostent consists of a 12 Fr body tapering to 8 Fr loops, and echogenicity measurements were performed with the loops. Additional equipment used included a Butterfly ultrasound device connected to an internet-enabled mobile phone, guide wires, sutures, syringes, and sterile water. These five stents were chosen based on their availability at our institution while ensuring the stent choices represented a variety of the current most commonly used stent materials (silicone and polyurethane) and stent sizes (6Fr and 8Fr).

**Table 1:** The 5 ureteral stents: brand, diameter, composition, and relative echogenicity with standard deviation (SD)

Brand	Diameter (Fr)	Composition	Relative Echogenicity
Cook Medical Black	6	Silicone	0.88 (± 0.11)
Bard Fluoro	6	Silicone	0.89 (± 0.05)
Coloplast Imajin Hydro	6	Silicone	0.91 (± 0.08)
Coloplast Stenostent	8	Silicone	1.04 (± 0.04)
Boston Scientific Ascerta	6	Polyurethane	0.76 (± 0.15)

### Procedure

Ultrasound has been used to quantify medical instruments with echogenicity in other specialties [14], and here we applied a similar principle to the ureteral stent in the field of urology. We standardized the ultrasound imaging setup by fixing the porcine bladder in an upright position on a board, ensuring no leaks. If leaks were detected, sutures were applied to seal them​. The bladder was filled with sterile water using a 250 ml beaker and syringe. Prior to insertion, the hydrophilic coating of the stents was activated by soaking in sterile water/saline. A guidewire was used to insert the stent into the bladder through its top opening. Once positioned, the guidewire was removed, and the stent was gently maneuvered using forceps under ultrasound and video-recorded to identify frames with optimal stent visibility for analysis.

The ultrasound recordings were taken using the Butterfly iQ + probe connected to the Butterfly cloud app. Each stent underwent three trials, and for each trial, video recordings were saved for analysis. We selected three frames from each trial for analysis. Using ImageJ software, we defined regions of interest (ROI) around each stent in each frame. We chose frames with best visibility, then used relatively fixed-size round ROI, centered on the most visible stent segment, avoiding artifacts and shadows, for consistent measurement. We measured the mean grey intensity within the ROI to quantify echogenicity, then normalized the average echogenicity of each stent against the Sensor wire. To minimize variability, the ultrasound gains and TGC settings were standardized across all experiments and were not altered during data collection. This ensured consistency in the imaging conditions for all stents.

We first compared the echogenicity of individual stents. Then, we organized stents into and compared within three groups based on materials and diameters: 6 Fr silicone, 8 Fr silicone, and 6 Fr polyurethane. Lastly, we performed T-tests to assess the significance between 6Fr silicone and 8Fr silicone stents and 6 Fr silicone and 6 Fr polyurethane stents.

## Results

### Placing ureteral stents under ultrasound guidance and quantifying their echogenicity is feasible

**Fig. 1:**
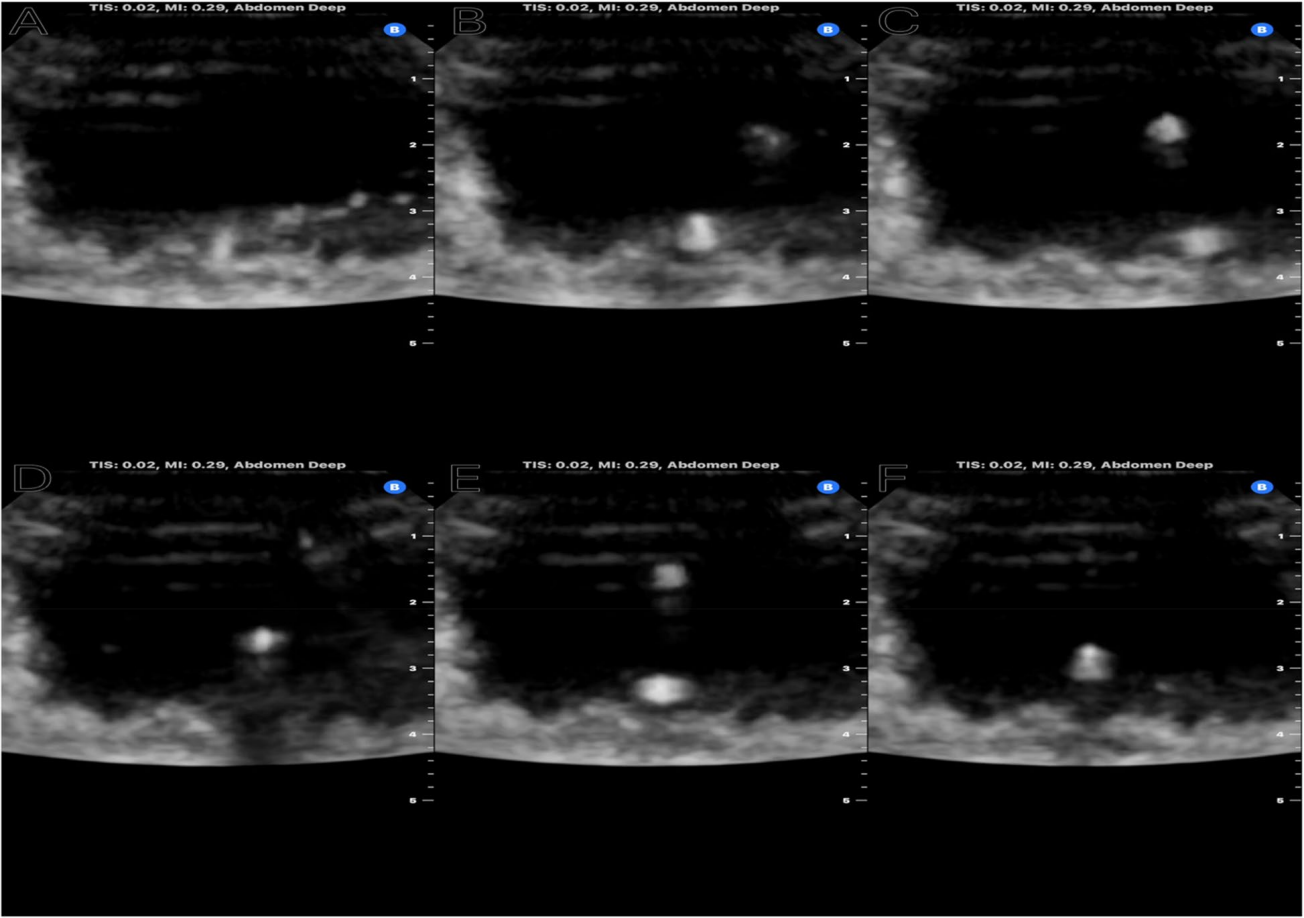
Ultrasound photographs of Sensor wire and five stents inside porcine bladders. **A**: Sensor wire. **B**: Cook Medical Black (6 Fr silicone). **C**: Bard Fluoro (6 Fr silicone). **D**: Coloplast Imajin Hydro (6 Fr silicone). **E**: Coloplast Stenostent (8 Fr silicone). **F**: Boston Scientific Ascerta (6 Fr polyurethane)

We began our study by testing the feasibility of placing ureteral stents under ultrasound guidance. We successfully visualized and recorded both static and dynamic images of the Sensor wire and each ureteral stent within a porcine bladder (Fig. 1). Additionally, we used ultrasound imaging to quantify the echogenicity of the ureteral stents. These results demonstrate that our approach is broadly applicable.

## Diameter is a more significant contributing factor to echogenicity than composition

**Fig. 2:**
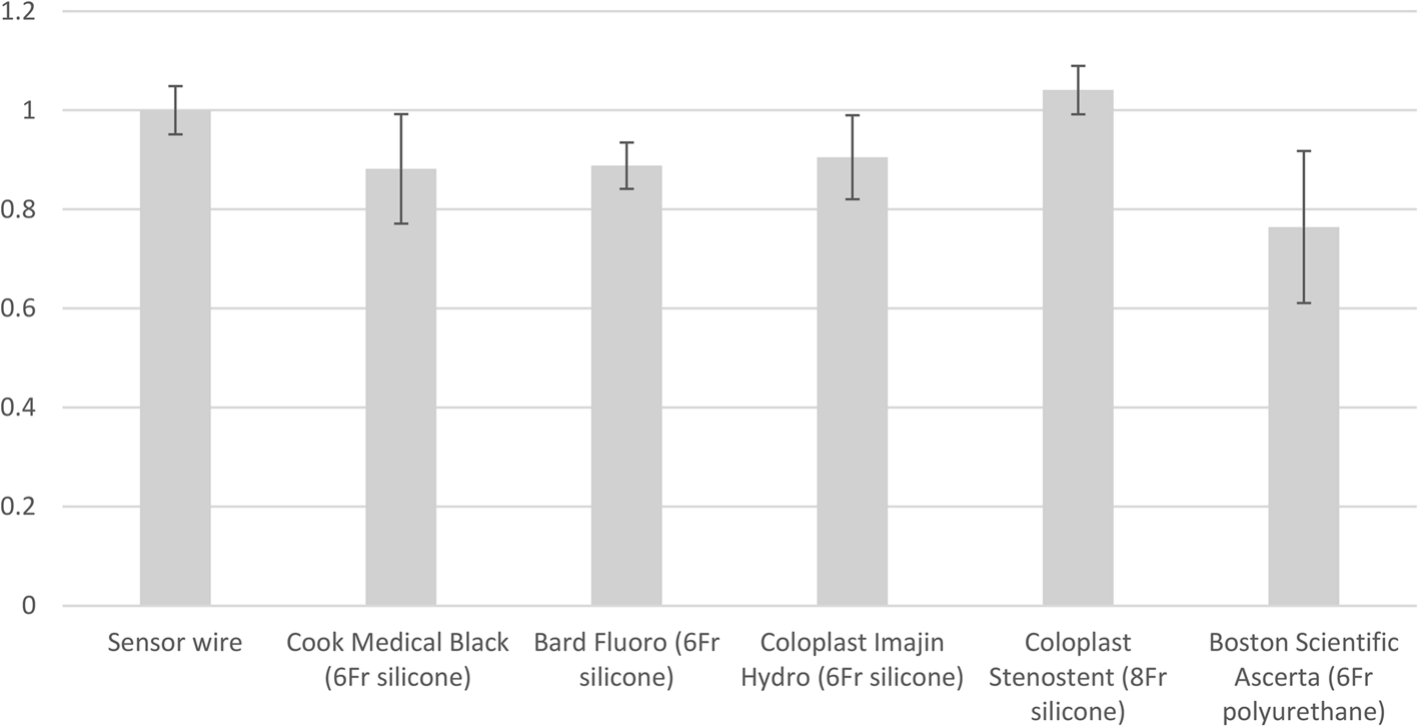
Relative Echogenicity by Stent Brand

We then calculated and compared the echogenicity of each ureteral stent relative to the Sensor wire. As shown in Fig. 2, we found that only the Coloplast Stenostent (8 Fr silicone) had a higher echogenicity value than the Sensor wire, at 1.04 (± 0.04). The other four stents exhibited lower relative echogenicity: Cook Medical Black (6 Fr silicone) at 0.88 (± 0.11), Bard Fluoro (6 Fr silicone) at 0.89 (± 0.05), Coloplast Imajin Hydro (6 Fr silicone) at 0.91 (± 0.08), and Boston Scientific Ascerta (6 Fr polyurethane) at 0.76 (± 0.15). These results indicate that stent diameter is the more important factor in measuring echogenicity; bigger diameter correlates with higher echogenicity.

## Silicone ureteral stent has higher relative echogenicity than polyurethane stent

**Fig. 3:**
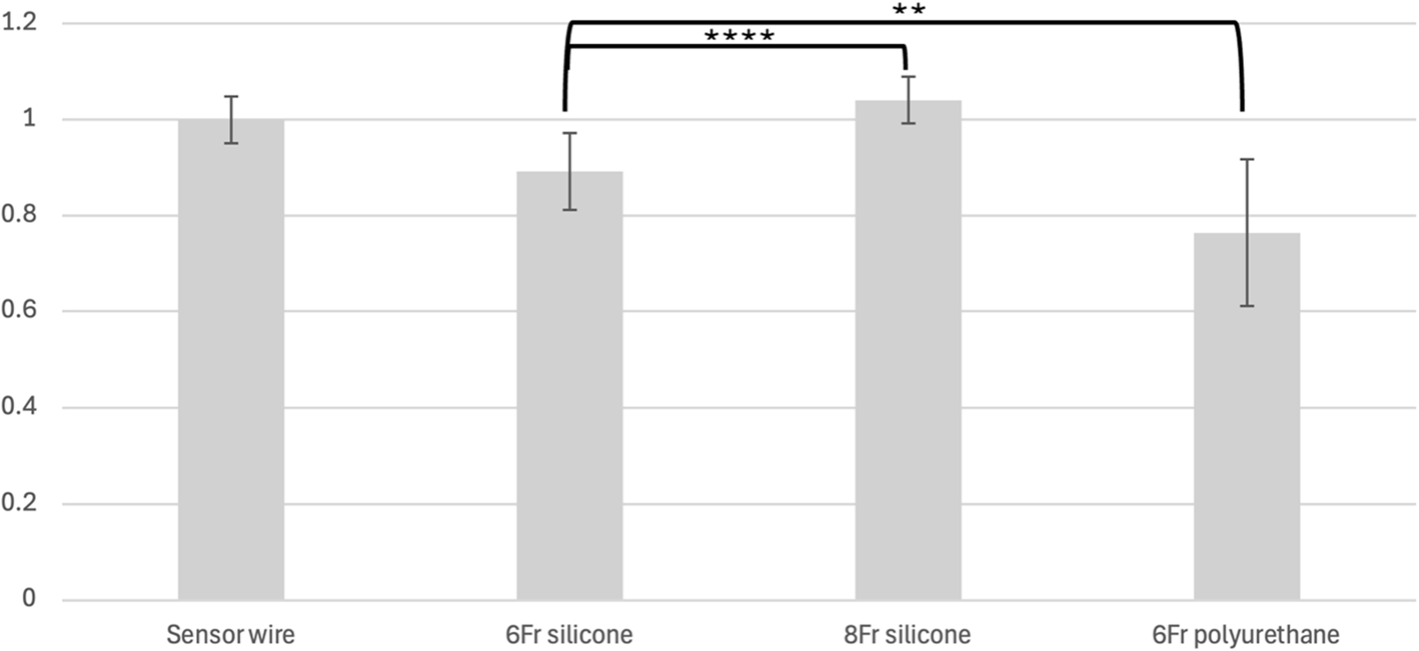
Relative Echogenicity by Stent Composition

We then grouped the ureteral stents into three groups based on diameters and compositions and compared their measured average echogenicity. The three groups were 8 Fr silicone, 6 Fr silicone, and 6 Fr polyurethane. We found that relative to the Sensor wire, only 8 Fr silicone stents had a higher echogenicity value at 1.04 (± 0.05). On the other hand, both 6 Fr silicone and polyurethane stents had lower relative echogenicity values, with 6 Fr silicone at 0.89 (± 0.08) and 6 Fr polyurethane at 0.76 (± 0.15). We showed in Fig. 3 that 8 Fr silicone stents were significantly more echogenic than 6 Fr silicone stents (*p* = 0.000012), and 6 Fr silicone stents were also significantly more echogenic than 6Fr polyurethane stents (*p* = 0.0029). Taken together, these results show that with the same diameter, silicone composition stent has relatively more echogenicity than polyurethane stent.

## Discussion

Ureteral stents are essential tools in urology but are associated with complications and frequent postoperative emergency visits [2, 3]. Although innovations in stent materials aim to improve patient outcomes are emerging [4–6], standardized methods for evaluating new stents remain limited. Our study, which is the first side-by-side comparison of stent echogenicity to the authors’ knowledge, demonstrates that ultrasound-based measurement provides a practical, radiation-free, and real-time method to assess stents.

First, we demonstrated that stents can be clearly visualized and analyzed in a porcine bladder model using ultrasound and image software. While our findings of increased echogenicity with larger stent diameters were not unexpected, we also observed significant differences in echogenicity depending on stent composition, which has not been described previously to the authors’ knowledge.

Taken together, these findings support the use of ultrasound as an alternative to fluoroscopy, especially for traditionally less radiopaque materials like silicone. This shift could be particularly impactful in reducing radiation exposure for both patients and healthcare providers during stent placement procedures. Given that silicone stents are often softer and more comfortable, enhanced ultrasound visibility may improve their utility in clinical settings.

While there are no prior comparative studies on this topic to the authors’ knowledge, these findings are further supported by clinical literature which has reported that silicone stents are associated with significantly lower pain scores compared to polyurethane stents [15, 16]. Another study demonstrated that silicone stents exhibited approximately 30% less encrustation in long-term in vitro models [17]. These observations may support the notion that echogenicity could correlate with procedural ease and patient comfort during ureteral stent insertion and removal.

Ureteral stents, arguably one of the most integral tools in urology, have been associated with complications since their invention, leading to ongoing innovations in their development and manufacturing. Our study employed a novel strategy to measure the echogenicity of ureteral stents, which may be helpful in the development of new stents and support a shift towards radiation-free technique. One limitation of our study is the lack of ureteral stents with new materials. While we focused on the most clinically-relevant and commonly used silicone and polyurethane stents, the inclusion of metallic and biodegradable stents in future studies could broaden our understandings. However, such new ureteral stents are not readily available in the market, so we chose to study the two most common materials currently (silicone and polyurethane) and set the baseline for future studies. Additionally, 5 Fr stents and 8 Fr polyurethane stents were not included in our study due to limited clinical relevance, with their use in adult urologic surgery limited to extenuating situations. With the emergence of new ureteral stent sizes and compositions and the increasing role of ultrasound in endourological procedures, the echogenicity of each stent should continue to be assessed in parallel with their development to ensure compatibility with current practice patterns. Moreover, the application of ultrasound for stent visualization may have further clinical utility, such as identifying calculi adjacent to the stent and placing stent during pregnancy, when minimizing radiation exposure is critical. While this study quantified differences in stent echogenicity, our ex vivo porcine bladder model did not replicate real-world variability, such as differences in patient habitus, bladder distention, or tissue attenuation, which can affect ultrasound visibility in clinical settings. Therefore, further in vivo research, ideally with stents of varying sizes within a single brand to control for compositional variability, is needed to determine whether these differences are perceptible in clinical practice and have a meaningful impact on procedural outcomes.

## Conclusion

Our study found that increased stent diameter and silicone composition (compared to polyurethane) are factors that lead to increased echogenicity within a porcine bladder model. This provides a novel comparative quantitative assessment of ureteral stent echogenicity, which can enhance sonographic diagnosis and guide the development of novel echogenic stents. Understanding the echogenic properties of stents is vital for the field of urology which is increasingly utilizing ultrasound guidance for endourologic procedures.

## Data Availability

The datasets generated and analyzed during the current study are available from the corresponding author (MM) upon reasonable request.
